# The Privileges of the Royal Colleges

**Published:** 1899-06-17

**Authors:** 


### THE PRIVILEGES OF THE ROYAL COLLEGES.

The fight between the Royal Colleges and the General Medical Council in regard to the right of the latter to exercise some control over the choice of teaching institutions in which "medical students" may pass the first of the five years of their medical curriculum is a matter of considerable importance to the medical profession, for it seems not unreasonable to believe that any lowering of the class of school from which medical students are recruited will in the end have an injurious effect upon the standard of general education prevailing in the medical profession. The root of the whole difference lies, of course, in the competition which is always going on between the rival examining and licensing bodies. These live by the examination fees paid by students, and the easier each qualifying body can make its curriculum the more students does it attract and the more fees flow into its coffers.

There can be no doubt that when it was decided some years ago that the medical curriculum should be lengthened, and that five years instead of four should elapse between registration as a medical student and qualification as a medical practitioner, the intention was that the extra year should be devoted to clinical work. What has happened, however, is that students have been permitted to spend the first year in the study of physics, chemistry, and elementary biology at certain recognised educational institutions other than medical schools. In principle this arrangement was right, for there seemed much reason to believe that at certain great colleges, or at educational institutions like those existing at South Kensington, it might be possible to arrange for better instruction on such subjects being given than would be possible at some of the smaller medical schools. The way in which the matter has worked out has, however, been by no means so satisfactory. Instead of the work of teaching these elementary and non-medical subjects being concentrated in great science schools, a host of little grammar schools, and even Board schools, have been "recognised," and thus schoolmasters, after getting their boys through the entrance examination as early as possible, have been able to keep them at school for another year doing elementary subjects; and these "schoolboy students" have been allowed to count that year as one of the five passed in medical study. It was evident that, as Sir Richard Thorne remarked, an attempt was being made "to whittle away the five years' system," and further, that the down grade competition between the colleges was leading to the recognition of schools and institutions of a standing quite different from what had been intended when the five years' course was instituted. In fact, the Council had ascertained that the Conjoint Board in England had recognised certain "teaching institutions" for the purposes of instruction, examination, and registration, without even the guarantee which inspection affords that such institutions are adequately equipped and efficiently conducted. Under these circumstances the committee of the Council, to whom the matter had been referred, proposed that no medical student should be registered until he had produced evidence that he had commenced medical study at some institution recognised by one of the licensing bodies *and approved by the Council*. This latter was the proviso that excited the indignation of the Royal Colleges. Sir Dyce Duckworth, speaking for the College of Physicians of London, said that "this was a new departure with a vengeance, and one which would destroy the autonomy of the Royal Colleges, who held their privileges by charter, and who meant to stand by these privileges whatever the Council might say or do." Brave words! But in the end the resolution was carried by 18 votes to six, five members declining to vote and one being absent.

